# Self-management interventions to patients with cirrhosis: A scoping review

**DOI:** 10.1097/HC9.0000000000000576

**Published:** 2024-11-04

**Authors:** Samsam Aden, Mette Munk Lauridsen, Lea Ladegaard Grønkjær

**Affiliations:** Department of Gastroenterology, University Hospital of Southern Denmark, Esbjerg, Denmark

**Keywords:** cirrhosis, interventions, liver disease, scoping review, self-efficacy, self-management

## Abstract

**Background:**

Self-management in chronic diseases like cirrhosis involves patients providing the necessary knowledge, skills, and confidence to enhance self-efficacy. This scoping review aims to describe the literature on self-management interventions in patients with cirrhosis to create an overview and identify key concepts and gaps in the existing literature.

**Methods:**

Four databases (CINAHL, Embase, Medline, and Scopus) were searched from November 2022 to September 2024. The review was reported in accordance with the Preferred Reporting Items for Systematic Reviews and Meta-Analysis extension for Scoping Reviews. Studies published from 2000 onward, including patients with cirrhosis of different etiology and severity, focusing on self-management and/or self-efficacy, and performed in a health care setting, were considered.

**Results:**

The search produced 1012 articles, of which 16 were included in the review. These represented studies from 7 countries and a sample of 1.276 patients. The studies differed in study design, sample size, delivery format, self-management interventions designed by the authors, and evaluation. However, all studies described some form of improvement in patient-related and clinical outcomes after the intervention, mainly improved patient knowledge and quality of life.

**Conclusions:**

Self-management interventions for patients with cirrhosis improved patient-related outcomes. However, more comprehensive and standardized interventions tailored to patients’ needs are needed. These self-management interventions should focus on increasing confidence and self-efficacy and address the different skills required by patients to manage their disease.

## Graphical abstract

**Figure:**
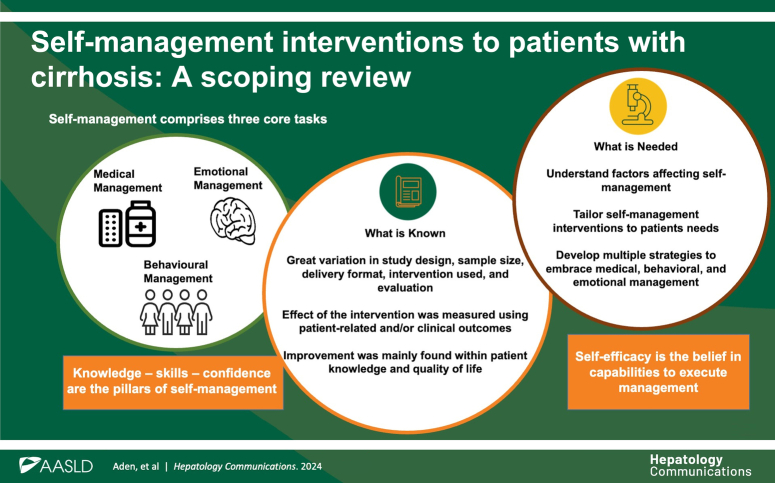

## INTRODUCTION

Cirrhosis is the end stage of all chronic liver diseases and a consequence of inflammation, which eventually leads to liver failure and fibrosis.^1^ The most common causes of cirrhosis include alcohol-associated liver disease, metabolic dysfunction associated with steatotic liver disease, and viral hepatitis. Cirrhosis is associated with high morbidity and mortality. Thus, it is the 11th most common cause of death, the third leading cause of death in people aged 45–64 years, and, together with liver cancer, accounts for 3.5% of all deaths worldwide.^2^

Intervention to treat cirrhosis consists of reducing further damage to the liver and treating secondary complications. However, treatment options are limited, and the only curative treatment is liver transplantation.^3^ Optimal management of cirrhosis can be challenging, as patients are required to follow complex medication regimens and dietary restrictions and engage in disease-monitoring activities. Chronic disease management is more effective if patients have the knowledge to manage their health.^4^ This notion aligns with new international recommendations that highlight the need to engage patients with cirrhosis in the care and treatment of their disease to increase self-management.^5^

Self-management refers to the patients’ ability to manage the lifestyle changes, symptoms, treatment and the mental, physical, and social impact of living with a chronic disease such as cirrhosis.^6^ Self-management can be viewed as a process where health care professionals, in interaction with the patient, can facilitate the patient with knowledge, skills, and confidence to enable patient activation to promote health and manage their disease, including knowing when and where to ask for support.^7^ Self-management skills are closely linked to self-efficacy, which covers the patients’ beliefs in their capabilities to manage specific problems.^8^ There are several studies of interventions developed to facilitate patients’ self-management in chronic diseases such as asthma, chronic heart disease, and diabetes. These studies have shown beneficial effects on patient knowledge, disease symptoms and/or burdens, self-efficacy, and quality of life.^9^^–^^11^ This highlights the need to implement such self-management interventions in liver disease care and treatment. However, to develop relevant and effective interventions, an overview is required to identify key concepts and gaps in the existing literature.^12^ This study, therefore, aimed to review the literature on self-management interventions in patients with cirrhosis.

## METHODS

A protocol for this scoping review was preregistered in the Open Science Framework (https://osf.io/registries), no. DOI 10.17605/OSF.IO/4ZUMB. The review was reported in line with the Preferred Reporting Items for Systematic Reviews and Meta-Analysis extension for Scoping Reviews.^13^

### Eligibility criteria

We included intervention studies with adults (over 18 y) with cirrhosis regardless of etiology and severity. No restrictions were imposed on the patient gender or ethnicity. Publications included in the review should mention self-management and/or self-efficacy in the description of the intervention, and the intervention should be performed in a health care setting. We included peer-reviewed full-text articles in English from the year 2000 and forward.

### Search strategy

The search strategy was based on a 3-step approach. First, Medline (Ovid) was searched with the search terms “cirrhosis” and “self-management,” followed by an analysis of the words contained in the titles and abstracts of the relevant articles and of the index terms used to describe the articles. Second, all 4 databases, CINAHL (Ebsco), Embase (Ovid), Medline (Ovid), and Scopus (Elsevier), were searched using all identified keywords and index terms from the first step in November 2022, July 2023, and September 2024. To refine the depth and width of the search and to capture available relevant articles, Boolean operators (OR and AND) were utilized to combine keywords and index terms such as patients with “cirrhosis” or “end-stage liver disease” and outcomes such as “self-management” or “self-efficacy.” Third, the reference list of all articles selected for critical appraisal and possible inclusion was manually searched for additional studies. The search strategy was reviewed by an experienced medical librarian and is presented in Table 1.

**TABLE 1:** Search strategy

The impact of self-management interventions in patients with cirrhosis		
Search terms: Self-management, self-efficacy, health education, patient education, empowerment, intervention studies, randomized controlled trial, quantitative research, cirrhosis, liver cirrhosis, end-stage liver disease, advanced liver disease, chronic liver disease		
Criteria’s		Databases
Inclusion	Exclusion	
**Study design**	Study design	Databases
• Intervention studies of different design • Randomized controlled trials	• Qualitative studies • Studies with no intervention • Systematic reviews or review studies	• Cinahl (via EBSCO) • Embase (via Ovid) • Medline (via Ovid) • Scopus (Elsevier)
**Language**		Manual search
• English		• Reference list of all selected articles
**Limits**		
• Human • Period 2000–2024		
**Patient population**		
• Patients with cirrhosis		
**Phenomena of interest**		
• Studies focusing on self-management and/or self-efficacy • Effect of self-management intervention • Intervention in health care setting		

### Article screening

Following the search, the articles were imported into a reference management program (Endnote X9, Clarivate Analytics, PA, USA), and duplicate citations were removed. Thereafter, the titles and abstracts of the articles identified from the searches were screened, and the selected articles were individually reviewed by the first and last authors. The full text of the articles selected was obtained and assessed for eligibility.

### Data extraction

Data were extracted from the included articles by the first and last authors. These data included details on the authors and country, year of publications, study design, number of participants, patient and disease characteristics, description of intervention, outcomes of significance to the review aim, and authors’ conclusion. At any step of the method phase, any disagreements were resolved through a discussion between the authors until a consensus was reached.

## RESULTS

**FIGURE 1:**
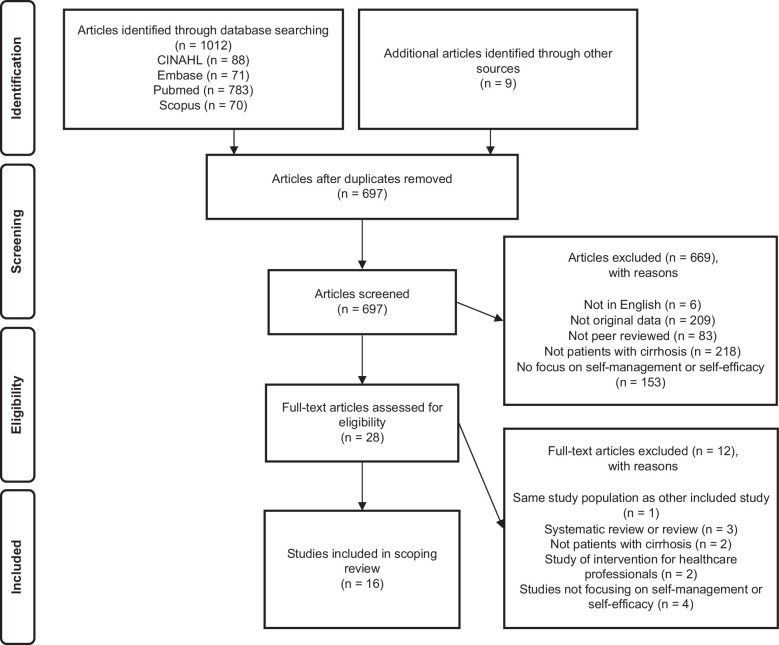
Flowchart of the review process.

The searches produced 1012 articles. After eliminating duplicates, 697 articles were reviewed based on titles and abstracts, and 28 were identified for full-text assessment. After full-text reviews, 16 articles met the inclusion criteria. The flowchart in Figure 1 summarizes the process.

### Description of the included articles

The included articles represented studies from 7 different countries across 4 continents, with 8 studies being from Asia (China, Iran, Japan, and Pakistan), 4 studies from Oceania (Australia), 2 studies from North America (USA), and 2 studies from Europe (England).^14^^–^^29^

A majority of the studies were randomized controlled trials^15^^,^^19^^,^^20^^,^^22^^,^^23^^,^^26^^,^^27^ followed by quasiexperimental studies,^14^^,^^25^^,^^28^ cross-sectional studies following an intervention,^17^^,^^21^^,^^24^ prospective studies,^16^^,^^29^ and a pilot study.^18^ The studies had a patient population size of 32–150. Eight of the studies had a control group for comparison between the intervention and usual care,^19^^,^^20^^,^^22^^,^^23^^,^^26^^–^^29^ while 2 studies compared 2 different interventions on 2 patient groups,^14^^,^^16^ and 6 studies compared the effect on the same patient group before and after an intervention.^15^^,^^17^^,^^18^^,^^21^^,^^24^^,^^25^

### Description of the included patients

A total of 1.276 patients participated in the included studies. The patient population consisted of 736 men (gender distribution reported in 15 studies),^14^^–^^20^^,^^22^^–^^29^ and the age ranged from 41 to 58 years (age reported in 15 studies).^14^^–^^20^^,^^22^^–^^29^ Eight studies had information on cirrhosis etiology. In these, a majority of patients had alcohol-associated liver disease (227 patients), followed by hepatitis B or C (229 patients), metabolic dysfunction–associated steatotic liver disease (88 patients), autoimmune or cholestatic liver disease (72 patients), other liver diseases (46 patients), or cryptogenic liver disease (32 patients).^14^^,^^17^^,^^19^^,^^20^^,^^24^^,^^26^^–^^28^ The severity of cirrhosis was reported in 6 studies, and the Child-Pugh score ranged between 7.5 and 10.0 and the MELD score between 8.0 and 19.0.^15^^,^^19^^,^^24^^,^^26^^,^^27^ Most studies included patients from various gastroenterology and hepatology outpatient settings, whereas 3 studies included inpatient. The characteristics of the studies and the patients are presented in Table 2.

**TABLE 2:** Data extraction from the included studies

References	Year	Type of study	No. participants	Liver disease category	Characteristics of participants	Intervention	Outcome	Author (s) conclusion
Alavinejad et al^14^ (Iran)	2019	Quasiexperimental study	72 patients with cirrhosis.	HBV (11%) HCV (44%) AIH (17%) NAFLD (6%) Others (22%)	• Mean age 47 y • 78% men • Mean MELD score 11.	Educational intervention and nutritional counseling were performed during 2-h visits. It included contents on liver function, cirrhosis, treatment, medications and management strategies, nutrition, healthy lifestyle advice, and dietary recommendations. At the end, a booklet with educational contents was given to the patients. Patients were followed via text messages and weekly calls. The intervention lasted 6 mo.	No difference before and after educational intervention in biochemical characteristics. Decrease in patients with ascites and edema after the intervention (*p*=0.005 and *p*=0.002). The days of hospitalization decreased after the intervention (1.28 vs. 0.33 d). Quality of life and knowledge score was increased after the intervention compared to before the intervention (4.22 vs. 7.11 and 141.89 vs. 182.72).	A simple education intervention can affect clinical outcomes, hospital admission days, quality of life, and knowledge of patients with cirrhosis. Further studies are warranted.
Bailey et al^15^ (USA)	2017	Randomized controlled trial	115 patients with cirrhosis awaiting transplantation. 56 received self-management training, and 59 received liver education.	Unknown	Self-management: • Mean age 56 y • 59% men • Mean MELD score 16 Liver education: • Mean age 56 y • 63% men • Mean MELD score 15	Either: Self-management intervention with six 30-min calls on coping skills training based on cognitive-behavioral principles and symptom management strategies. Or: Six 30-min calls with education sessions about liver functions, disease knowledge, and management.	No difference between groups after 10 and 12 wk postbaseline based on outcomes such as illness uncertainty (86.1 vs. 88.8), depressive symptoms (9.9 vs. 9.8), anxiety (4.9 vs. 4.7), uncertainty management (78.0 vs. 78.5), self-efficacy (62.5 vs. 63.7), and quality of life (72.3 vs. 72.9).	Both groups improved self-efficacy. Self-management group (62.5–66.8). Liver education (63.7–64.9) This study offers insight regarding future interventions.
Beg et al^16^ (England)	2016	Prospective study	39 patients with cirrhosis.	Group A: ALD (70%) NASH (10%) PBC (5%) AIH (10%) VH (5%) Group B: ALD (63%) NASH (16%) PBC (11%) VH (5%) HC (5%)	Group A: • Mean age 59 y • 75% men • Child-Pugh Score ∘ A (50%) ∘ B (30%) ∘ C (20%) Group B • Mean age 56% • 74% men • Child-Pugh Score ∘ A (47%) ∘ B (37%) ∘ C (16%)	Two-page patient information leaflet explaining the diagnosis of cirrhosis and its potential complications was provided to the patients. Group A patients were telephoned by a single junior doctor to go through a questionnaire to assess patients’ knowledge and understanding of liver cirrhosis prior to receiving the leaflet. Both groups were telephoned after 2 months to answer the questions again.	No difference between group A or group B. Only 35% of patients could give a meaningful definition of cirrhosis and were able to identify complications of cirrhosis. Patients who had read the leaflet had higher questionnaire score (4 vs. 7.5) compared to those, who had not read the leaflet.	There was a poor level of basic understanding of cirrhosis and its complications. The introduction of a leaflet resulted in an improvement in understanding. Longitudinal studies are required.
Goldsworthy et al (England)^17^	2017	Cross-sectional study followed by intervention	52 patients with cirrhosis.	ALD (37%) VH (19%) NAFLD (17%) CLD (17%) Other (10%)	• Mean age 56 y • 62% men	Educational intervention with 12-min long video explaining the functions of the liver, the development of cirrhosis, the complications, and the associated management strategies. A questionnaire to assess patient knowledge of the complications and management of liver cirrhosis at baseline and at least 1 mo after viewing the video was distributed to the patients.	Prior the video intervention, only 25% of the questions were answered correctly. One month after the intervention, 67% were answered correctly.	Patients had poor baseline knowledge of cirrhosis. Delivering information by video led to improvement. This is an effective way to empower patients.
Hayward et al^18^ (Australia)	2017	Pilot study	50 patients with decompensated cirrhosis.	Unknown	• <60 y (60%) • 78% men	Educational intervention in the form of a chronic disease booklet describing complications of liver disease provided information about dietary modifications and medications and space to document medications, appointments, weight, and blood pressure.	Before intervention: 54% of patients could recall being given written information by a clinician, and 64% had self-sought information. Three months after intervention: 85% reported it was helpful, and 78% used it in between clinical appointments. Mean of 8.1 correct answers of 13 in recall questionnaire to examine information retention from the booklet.	Implementation and evaluation of educational tools specific to the learning needs of local patients with chronic disease may improve patient engagement in self-management.
Hayward et al^19^ (Australia)	2020	Randomized controlled trial	116 patients with decompensated cirrhosis. 57 patients in intervention group and 59 patients in usual care group.	Intervention: ADL (39%) HVC (37%) NAFLD (14%) Other (11%) Usual care: ADL (58%) HVC (29%) NAFLD (10%) Other (3%)	Intervention: • Mean age 58 y • 68% men • MELD median score 14.5 • Mean Child-Pugh score 8 • Child-Pugh Score ∘ A (14%) ∘ B (64%) ∘ C (21%) Usual care: • Mean age 59 y • 63% men • MELD median score 12.5 • Mean Child-Pugh score 7.5 • Child-Pugh Score ∘ A (31%) ∘ B (55%) ∘ C (14%)	Up to 4 contacts over a 6- to 8-mo period from a trained clinical pharmacist in person or via telehealth in addition to usual care. Patients received disease and medication education and reactive advice tailored to the patients’ individual needs.	Intervention patients compared to usual care patients: Improved correct responses to knowledge and self-care questions regarding medication (5–6 vs. 4–4). Greater self-perceived understanding of liver disease and self-reported quality of life (3.9–4.3 vs. 4.0–4.1).	Resources are needed to support implementation of evidence-based measures at local centers to improve patient knowledge.
Huang and Deng^20^ (China)	2020	Randomized controlled trial	112 patients with cirrhosis. 56 patients in intervention group and 56 patients in usual care group.	Intervention: VH (39%) ALD (25%) TLC (16%) PBC (9%) Other (11%) Usual care: VH (41%) ALD (21%) TLC (14%) PBC (11%) Other (13%)	Intervention: • Mean age 44 y • 68% men Usual care: • Mean age 45 y • 59% men	During hospitalization, patients received intervention and guidance in psychology, diet, sleep, medication, and other things. When necessary, the patient was referred to a psychologist. After discharge, weekly telephone follow-up was conducted. Both groups received health education at hospitalization. The content included explanation of cirrhosis, the patients’ self-psychological adjustment, diet and sleep condition, prevention of infections, and exercise. Health education books were issued to the patients at discharge. The groups were followed for 7 mo.	Intervention patients compared to usual care patients: Increase in medical compliance (87.5 vs. 66.7), decrease in anxiety (65.12–50.23 vs. and 66.02–39.34) depression (64.21–51.4 vs. 63.21–44.23), improved quality of life: Physiological function (46.97–69.54 vs. 45.43–62.78), social function (47.12–73.45 vs. 46.85–65.16), Mental state (47.51–72.18 vs. 47.04–64.96), energy (46.20–71.82 vs. 45.34–63.80)	Results showed improvement in medication compliance, self-efficacy, and quality of life in intervention group. The finding provides evidence-based guidance.
Kadokawa et al^21^ (Japan)	2017	Cross-sectional study followed by intervention	49 patients with cirrhosis	Unknown	No information	Liver disease education, focusing on treatment and prevention of liver disease and hepatic cancer, diet restriction, and the importance of branched-chain amino acid preparations. The knowledge levels of the patients were evaluated semiquantitatively using a four-point scale. The questionnaire was administered immediately before and after class attendance.	The knowledge level of the patients improved with class attendance, and the degree of these improvements differed according to the number of class attendances. Those who had attended the education class zero to 2 times showed improvement in disease knowledge on prohylaxis of hepatic cancer (0.76 and 1.34), treatment of hepatic cancer (0.91 and 1.14), iron restriction (0.54 and 1.13), and effects of branched-chain amino acid (0.32 and 0.78)	The results indicate that liver disease education is effective in improving the knowledge of patients.
Li and Chen^22^ (China)	2020	Randomized controlled trial	104 patients with ascites caused by hepatitis B–related cirrhosis. 52 patients in intervention group and 52 patients in usual care group.	HBV (100%)	Intervention: • Mean age 52 y • 63% men Usual care: • Mean age 53 y • 58% men	Empowerment education is divided into 4 steps: Question determination, emotional expression, goal setting, plan confirmation, and behavior evaluation combined with nutritional care. Conducted within 6 h after admission for 20 min and repeated every 3–5 d until discharge.	Intervention patients compared to usual care patients: Increased self-management behavior (58.73–83.98 vs. 58.85–73.14), decrease in anxiety (57.07–39.32 vs. 56.87–46.95) and depression (44.59–31.90 vs. 45.23–40.77) score, and higher nutritional indices (increased upper arm circumference, upper arm muscle circumference, triceps skin fold thickness, body mass index, albumin, prealbumin, and hemoglobin).	An empowerment education model combined with nutritional care can effectively improve the self-management, relieve anxiety and depression, and improve nutritional status.
Mansouri et al^23^ (Iran)	2017	Randomized controlled trial	74 patients with cirrhosis. 37 patients in intervention group and 37 patients in usual care group.	Unknown	Intervention: • Mean age 42 y • 65% men Usual care: • Mean age 41 y • 70% men	Six 90-min sessions twice a week containing information of cirrhosis, diet, medication, problem-solving, decision-making, cognitive-behavioral techniques, and empowerment of positive attitude. Muscle relaxation audio CDs were also given to the patients. Patients was followed for a month via telephone.	Intervention patients compared to usual care patients: Total score (74.62–102.24 vs. 76.78–76.78) and the scores of all the subscales of self-efficacy increased (stress reduction 25.05–35.59 vs. 26.68–26.15, decision-making 8.81–12.32 vs. 7.60–7.36, and Positive attitude 40.75–54.32 vs. 42.50–43.26)	Self-management program resulted in improvement of self-efficacy in patients with cirrhosis. Supportive strategies could be useful to improve care and prevent complications.
Volk et al^24^ (USA)	2013	Cross-sectional study followed by intervention	150 patients with cirrhosis.	ADL (16%) VH (48%) NAFLD (19%) Other (17%)	• Mean age 57 y • 59% men • Median MELD score 8 • Child-Pugh Score ∘ A (70%) ∘ B (27%) ∘ C (3%)	Survey to test disease self-management knowledge. Education using a booklet covering prevention and management of cirrhosis as well as topics such a surgery and hospitalization. Patient were to keep track of their medications, appointments, and weight. Three months later, the patients received a repeat survey.	Prior to the booklet intervention, only 53% of the questions were answered correct. After the intervention, 67% were answered correct.	Patients with cirrhosis lack important knowledge about disease self-management. Knowledge was improved by a simple educational intervention. Further studies are needed to determine whether more intensive educational interventions can effectively and cost-effectively improve outcomes in patients with cirrhosis.
Waris et al^25^ (Pakistan)	2022	Quasiexperimental study	32 patients with decompensated cirrhosis.	Unknown	• 19% age between 21 and 40 y • 81% aged between 41 and 60 y • 47% men	Educational intervention using educational sessions based on patients’ level of understanding. It included disease, complications, diet, medication, knowledge of warning signs, and how to self-manage disease. In addition, booklet was handed to patients. Intervention lasted for 12 wk. A questionnaire to assess patient knowledge and quality of life was distributed.	Prior to the intervention, the median knowledge score regarding self-management was 4. After the intervention, it was increased to 10 The quality of life score was 54.5 before the intervention and 114 after the intervention.	The educational intervention may have a considerable positive effect on self-management skills and quality of life.
Wigg et al^26^ (Australia)	2013	Randomized controlled trial	60 patients with decompensated cirrhosis. 40 patients in intervention group and 20 patients in usual care group.	Intervention: ALD (35%) HCV (10%) NAFLD (2.5%) AIH (2.5%) Usual care: ALD (60%) HCV (10%) NAFLD (10%) AIH (5%)	Intervention: • Mean age 53 y • 72% men • Mean MELD score 11 • Mean Child-Pugh score 9.3 Usual care: • Mean age 52 y • 55% men • Mean MELD score 10.5 • Mean Child-Pugh score 9.1	Four chronic disease management components: Delivery system design (multidisciplinary team care, home visits and weekly nurse telephone reviews), decision support (using evidence-based protocols), self-management support (education concerning diet, medications, and need for investigations), clinical information systems. Intervention lasted for 12 mo.	Intervention patients compared to usual care patients: Higher rate of attendance at outpatient care (IRR 1.3; 95% CI: 1.1–1.6) and increase in quality of care (HCC screening 100% vs. 89%, referral for liver transplant assessment 18% vs. 0%, commencement of hepatitis A and B vaccination 91% vs. 11%, performance of bone density 98% vs. 75%, and vitamin D testing 87% vs. 59%). No difference in hospital admission rates (IRR 2.2, 95% CI: 1.0–4.5), improved quality of life (3.4–4.0 vs. 3.1–3.7), disease severity (MELD score 12.2–11.8 vs. 14.0–10.1, Child-Pugh score 9.3–8.3 vs. 9.1–7.8) risk of death (HR 0.6, 95% CI: 0.3–1.5).	Larger trials with longer follow-up periods are required to confirm findings and assess cost-effectiveness.
Wigg et al^27^ (Australia)	2024	Randomized controlled trial	147 patients with decompensated cirrhosis. 75 patients in intervention group and 71 patients in usual care group.	Intervention: ALD (68%) HCV (12%) NASH (17%) AIH (1%) CLD (1%) Usual care: ALD (69%) HCV (10%) NASH (16%) AIH (1%) CLD (1%) Other (3%)	Intervention: • Mean age 56 y • 72% men • Mean MELD score 19 • Mean Child-Pugh score 10 Usual care: • Mean age 54 y • 63% men • Mean MELD score 18 Mean Child-Pugh score 9	The intervention was based on chronic disease management principle. Four chronic disease management components: delivery system design (home visits, phone calls, rapid access to care pathway, reminders for appointments), self-management support (patient information booklet, action plans, medication blister packs, enhanced participant support and education), clinical information systems (participant data sheet, automated recall and reminder system).	Intervention patients compared to usual care patients: No difference in overall admission rate, but lower encephalopathy admission rate (HR 1.87, 95% CI: 1.18–2.96). Higher rate of elective versus emergency admissions (IRR 1.42, 95% CI: 1.10–1.83). No difference in survival (HR 1.14, 95% CI: 0.66–1.96). Increase in quality of care with performance of bone density 75% vs. 41%, vitamin D testing 80% vs. 45%, and HCC surveillance adherence 67% vs. 51%. No difference in knowledge, self-management ability, (75.9 vs. 73.05), barriers to medication adherence (20.1 vs. 22.0) quality of life (4.06 vs. 3.97) and (0.51 vs. 0.44) but difference in VAS-scale (68.74 vs. 57.77)	The chronic disease management intervention did not reduce overall admission rate events and may not be effective in decompensated cirrhosis.
Zandi et al^28^ (Iran)	2005	Quasiexperimental study	44 patients with cirrhosis. 21 patients in intervention group and 23 patients in usual care group.	Intervention: HBV (45%) HVC (20%) AIH (20%) Other (20%) Usual care: HBV (50%) HVC (15%) AIH (20%) Other (20%)	Intervention: • Mean age 40 y • 50% men • Child-Pugh score ∘ A (20%) ∘ B (50%) ∘ C (30%) Usual care: • Mean age 46 y • 70% men • Child-Pugh score ∘ A (20%) ∘ B (40%) ∘ C (40%)	The education program consisted of education within the nature of liver disease, coping strategies in systemic symptoms, worry, and depression, relaxation techniques, diet and nutrition, and medical therapies. The intervention group received education in groups together with their relatives. Each education session lasted for 45 min. Posters, slides, and manikin were also used to facilitate the learning process, and pamphlets were given to the patients. Phone calls every 2 wk. The intervention lasted 3 mo.	Intervention patients compared to usual care patients: No difference in quality of life before the intervention. After the intervention, an increase in quality of life in the intervention group and decrease in quality of life in the usual care group (139–171.9 vs. 137–112.5).	The results confirm the positive effects of an educational and self-care program on quality of life in patients with cirrhosis. Further studies with extensive programs and with long-term follow-up are suggested.
Zhang et al^29^ (China)	2019	Prospective study	60 patients with cirrhosis. 30 patients in intervention group and 30 patients in usual care group.	Unknown	Intervention: • Mean age 62 y • 50% men Usual care: • Mean age 61 y • 57% men	Four-stage intervention using the principles of health empowerment theory: analyze of personal and lifestyle characteristics, nurses helped the patients and their families to understand the risk factors for cirrhosis and discussed the importance of diet, exercise, medication, etc., nurses discussed self-management skills with the patients.	Intervention patients compared to usual care patients: Increased understanding of clinical symptoms, etiology, diet and nutrition, use of medication, treatment, and disease awareness (82–242 vs. 84–166). Improvement in activity of daily living (80.5 vs. 68.5) and increased health-promoting lifestyle profile (147.6–159.2 vs. 116.3–125.6).	Health education guided by patient empowerment theory was beneficial to the active rehabilitation of patients with liver cirrhosis and improved quality of life.

Abbreviations: AIH, autoimmune hepatitis; ALD, alcohol-associated liver disease; CLD, cholestatic liver disease; HC, hemochromatosis; IRR, internal rate of return; PBC, primary biliary cirrhosis; TLC, toxic liver disease; VAS, visual analogue scale; VH, viral hepatitis.

### Delivery format of the interventions

The interventions were most commonly delivered in an outpatient location^14^^–^^19^^,^^21^^,^^23^^–^^28^ followed by inpatient^22^^,^^29^ and mixed.^20^ Most studies had multiple contacts/visits with the patients during the intervention,^14^^,^^15^^,^^19^^,^^20^^,^^23^^,^^25^^–^^29^ while 5 studies only had 1.^16^^–^^18^^,^^21^^,^^24^ Two studies delivered the intervention in-person (ie, psychical lectures, provided instructions, and/or support),^21^^,^^22^ 6 studies delivered remotely (ie, booklet handed out, telephone, text messages, video, or self-monitoring)^15^^–^^19^^,^^24^ and 7 studies used both methods.^14^^,^^20^^,^^23^^,^^25^^–^^29^ The studies delivered the intervention on an individual basis^14^^–^^18^^,^^20^^,^^22^^,^^24^^–^^27^^,^^29^ in a group setting,^21^^,^^23^^,^^28^ or a combination of both.^19^ In 4 studies, nurses were responsible for providing the intervention,^20^^,^^22^^,^^25^^,^^28^^,^^29^ while 4 studies used a multidisciplinary approach, including nurses, hepatologists or gastroenterologists, and other allied health professionals.^14^^,^^15^^,^^19^^,^^26^^,^^27^ In 4 studies, there was no involvement of health care professionals besides delivering a booklet or showing a video^16^^–^^18^^,^^24^ and in 2 studies, it was unclear who delivered the intervention^21^^,^^23^ (Table 3).

**TABLE 3:** Delivery and evaluation of interventions

Characteristics	No. studies, n (%)
Location	
Outpatient	13 (81)
Inpatient	2 (13)
Mixed	1 (6)
In-person/remote delivery	
In-person (education, instructions, supervision)	2 (13)
Remote (booklet handed out, telephone, video, self-monitoring)	6 (38)
Mixed	8 (50)
Group/individual delivery	
Individual	12 (75)
Group	3 (18)
Mixed	1 (6)
Materials used	
Printed educational materials	14 (88)
Verbal instructions (nature of cirrhosis, empowerment, cognitive-behavioral principles, symptom management strategies, educational sessions, problem-solving, decision-making, diet)	8 (50)
Digital materials	5 (13)
Unclear	1 (7)
Health care professionals involved	
Nurse	8 (53)
Hepatologist/gastroenterologist	3 (20)
Other allied health professionals (alcohol counselors, clinical pharmacist, dietician, general practitioner, social worker)	4 (27)
None (booklet handed out or video shown)	4 (27)
Unclear	2 (13)
Setting where intervention are delivered	
Educational program	10 (63)
Self-management program	3 (18)
Chronic disease management program	2 (13)
Self-care program	1 (6)
Evaluation of intervention	
Use of validated questionnaires (to assess anxiety, depression, medication adherence, self-efficacy, self-management, and/or quality of life)	11 (69)
Use of self-made questionnaires to assess increased knowledge	10 (63)
No. hospitalizations	3 (18)
Disease severity/presence of complications	2 (13)
Planned outpatient contact	2 (13)
Biochemical characteristics	2 (13)
Survival	1 (6)
Nutritional status	1 (6)

### Description of the interventions

Ten studies described the intervention as a form of education program,^14^^,^^16^^–^^22^^,^^24^^,^^29^ 3 as a self-management program,^15^^,^^23^^,^^25^ and 2 as a chronic disease management program^26^^,^^27^ or a self-care program.^28^ The intervention programs were all designed for the purpose of the studies. One study included a patient representative in the intervention design.^17^ Some studies described that the interventions were designed based on reviewing past and recent literature,^25^ an empowerment model,^22^ empowerment theory,^29^ multifaceted intervention supported by evidence,^26^^,^^27^ and uncertainty in illness theory.^15^ All of the studies used patient education material in the form of either booklets, psychical lectures, or videos covering various topics such as explaining the function of the liver, the diagnosis of cirrhosis, its potential complications, and providing information on dietary and medication to facilitate self-management.^14^^–^^29^ In 3 studies, it was described that the intervention was tailored to the patients’ needs.^19^^,^^22^^,^^28^ Seven studies focused exclusively on increasing patients’ knowledge.^14^^,^^16^^–^^19^^,^^21^^,^^24^ Eight studies supplemented the education with other interventions such as behavior control, cognitive-behavioral techniques, decision-making, emotional expression, empowerment education, goal setting, problem-solving, referral to a psychologist, relaxation techniques, symptom management training, and specific patient action plans to increase skills and confidence in self-management and self-efficacy, manage physical symptoms, and provide psychological support to the patients^15^^,^^20^^,^^22^^,^^23^^,^^25^^–^^29^ (Table 4).

**TABLE 4:** Description and effects of the interventions

Type of intervention	Description	No. studies (%)
Patient education	Educational sessions or material covering various topics such as explaining the diagnosis of cirrhosis and its potential complications, providing information about dietary and medications aiming at facilitating self-management	16 (100)
Patient empowerment and coping strategies	Information, support, and advise to facilitate management of the disease and daily living aiming at increasing patient empowerment and coping strategies in connection with the liver disease	7 (44)
Managing physical symptoms	Strategies and support to facilitate management of physical symptoms such as ascites (including information of medication, self-monitoring of weight, sodium restriction, preventive measures, and emergency procedures)	3 (19)
Psychological support	Support for managing psychological well-being (including psychological counseling)	1 (6)
Effect of the intervention	Description	
Questionnaires	Increased knowledge score	7 (44)
	Increased quality of life	5 (31)
	Decreased anxiety and depression	2 (13)
	Increased self-management behavior	2 (13)
	Increased self-efficacy	1 (6)
	Improved activity of daily living	1 (6)
	Improved health promotion	1 (6)
	Increased self-perceived understanding	1 (6)
Other outcome measure	Increased quality of care	2 (13)
	Increased attendance at the outpatient clinic	1 (6)
	Increased elective hospitalizations	1 (6)
	Increased medical compliance	1 (6)
	Increased nutritional status	1 (6)
	Decrease in ascites and edema	1 (6)

### Evaluation of the interventions

In 13 studies, the interventions were evaluated after 1–24 months.^14^^–^^20^^,^^23^^–^^29^ One study evaluated the intervention immediately after a lecture with patient education,^21^ and 1 study evaluated the intervention in connection with patients being discharged from the hospital.^22^

All studies reported quantitative patient-related and/or clinical outcome measures in connection with the evaluation. In 9 studies, self-made questionnaires were developed to assess knowledge (6 studies)^14^^,^^16^^–^^19^^,^^21^^,^^29^ or self-management behavior (2 studies)^22^^,^^25^ of the patient’s preintervention and postintervention. Eleven studies used validated questionnaires to assess the effect of the intervention on patients’ activity of daily living and health promotion, anxiety, depression, medication adherence, self-efficacy, and/or quality of life.^14^^,^^15^^,^^19^^,^^20^^,^^22^^,^^23^^,^^25^^–^^29^ Four studies reviewed biochemical characteristics, disease complications and severity, nutritional status, number of hospitalizations and planned outpatient visits, and quality of care before and after the interventions.^14^^,^^22^^,^^26^^,^^27^

### Effects of the interventions on patient-related outcomes

Seven studies demonstrated improved patient knowledge regarding cirrhosis disease and management measured by different self-made questionnaires^14^^,^^16^^–^^19^^,^^21^^,^^29^ and improved self-management behavior (58.73–83.98 and 4.0–10.0) also measured by self-made questionnaires.^22^^,^^25^ One study found no improvement in self-management ability between the intervention and control group.^27^

One study found improvement in activity of daily living in the intervention group compared to the control group measured by the Barthel Index (80.5 vs. 68.5) and health promotion measured by the Health Promoting Lifestyle Profile II (159.2 vs. 125.6) 2 months after discharge.^28^

Two studies found decreased anxiety and depression in the intervention groups compared to the control group measured by the Self-Rating Anxiety Scale (65.12–50.23 vs. 66.02–39.34 and 57.07–39.32 vs. 56.87–46.95 ) and the Self-Rating Depression Scale (64.21–51.4 vs. 63.21–44.23 and 44.59–31.90 vs. 45.23–40.77).^20^^,^^22^

One study found an increase in medical adherence in the 2 groups after the intervention (87.5 vs. 66.7) using a self-made compliance evaluation scale,^20^ while another study did not find any difference in barriers to medication adherence.^27^

One study found increased self-efficacy after the intervention compared with usual care (74.62–102.24 vs. 76.78–76.78),^23^ while another study found an improvement in self-efficacy between the intervention and control groups.^15^ One study showed a greater self-perceived understanding of liver disease at follow-up compared with usual care.^19^

Six studies showed improved quality of life.^14^^,^^19^^,^^20^^,^^25^^,^^27^^,^^28^ Four studies used the Chronic Liver Disease Questionnaire and showed an improvement (from 4.22 to 7.11 and 54.5 to 114.0) in patients preintervention and postintervention,^14^^,^^25^ and an improvement in the intervention group as opposed to the control groups (3.9–4.3 vs. 4.0–4.1 and 139.0–171.9 vs. 137.0–112.5).^19^^,^^28^ One study found no difference with the Chronic Liver Disease Questionnaire but a difference between the intervention and control group using the EuroQol visual analog scale (54.96–68.74 vs. 53.67–57.77).^27^ One study used the Short-Form 36 and showed an improvement between the groups (only items scores presented; Table 2). Two studies found no improvement in quality of life after the intervention compared with control groups^15^^,^^26^ (Table 4).

### Effects of the interventions on clinical outcomes

One study found no difference in biochemical characteristics in the patients before and after the intervention. The same study found a decrease in the presence of ascites and edema in the patients 6 months after the intervention but no difference in the presence of HE or variceal bleeding.^14^ Another study found no difference in disease severity (Child-Pugh score 9.3–8.3 vs. 9.1–7.8 and MELD score 12.2–11.8 vs. 14.0–10.1) or risk of death (HR 0.6, 95% CI: 0.3–1.5).^26^

One study found improved nutritional status in the intervention group compared with the control group measured by upper arm (23.54–26.94 vs. 23.37–25.50 cm), muscle circumference (15.35–21.21 vs. 15.01–18.87 cm), triceps skin fold thickness (11.02–13.88 vs. 10.37–12.08 cm), body mass index (17.33–23.22 vs. 17.45–20.37), albumin (28.49–36.71 vs. 28.60–33.52 g/L), prealbumin (0.19–0.33 vs. 0.18–0.26 g/L), and hemoglobin (127.02–130.7 vs. 82.62–110.75 g/L).^22^

One study found a decrease in hospitalization (from 1.28 to 0.33 d) over a period of 6 months.^14^ Two other studies found no difference in overall hospital admission rates between the intervention and control groups but an 87% lower HE admission rate, a 42% increase in elective versus emergency admissions, and a 30% increase in attendance to the outpatient clinic.^26^^,^^27^ These studies also found improved quality of care measured by increase in HCC screening (100% vs. 89% and 67% vs. 51%), referral for liver transplant assessment (18% vs. 0%), commencement of hepatitis A and B vaccination (91% vs. 11%), performance of bone density (98% vs. 75% and 75% vs. 41%), and vitamin D testing (87% vs. 59% and 80% vs. 45%) between the intervention and control group.^26^^,^^27^

## DISCUSSION

This scoping review presented an overview of the literature on self-management interventions for patients with cirrhosis to identify key concepts and gaps in the existing literature. The review included 16 studies from different countries, including patients with cirrhosis of different etiology and severity. The studies varied in study design, sample size, delivery format, self-management interventions designed by the authors, and evaluation. However, all described some improvement in patient-related or clinical outcomes after the intervention, mainly within improved patient knowledge and quality of life.

Former studies have found that the level of self-management is moderate in patients with liver disease, although comparison is difficult because of different self-management scales.^30^^,^^31^ Thus, focusing on increasing patients’ self-management is relevant to provide the patients with knowledge, skills, and confidence to enable patients to take a more active role in their health and improve patient-related and clinical outcomes.^5^^,^^32^

**FIGURE 2:**
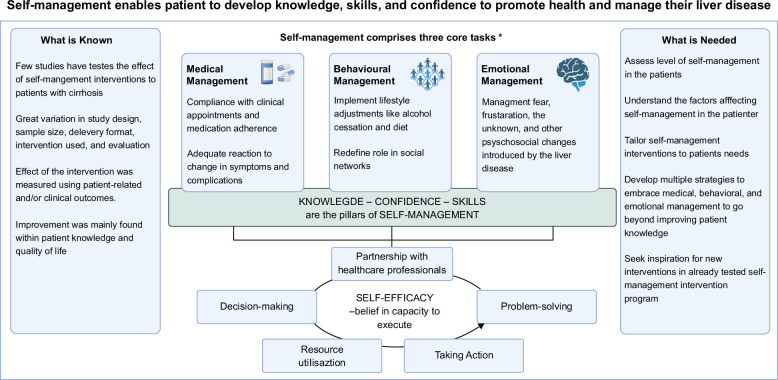
Overview of self-management findings and needs. Adapted from Corbin and Strauss.^34^

The term self-management is used widely and is described by various definitions, contributing to a lack of agreement and clarity in the literature.^33^ This may also reflect the differences in the interventions in the included studies. Initially identified by Corbin and Strauss, self-management comprises 3 core tasks (medical management, behavioral management, and emotional management). These tasks were later underpinned by 5 skills (decision-making, partnership with health care professionals, problem-solving, resource utilization, and taking action) the patients should possess. Self-efficacy is required to engage and execute the set of tasks and skills^34^ (Figure 2).

In this scoping review, 7 studies focused exclusively on increasing patients’ knowledge.^14^^,^^16^^–^^19^^,^^21^^,^^24^ Knowledge about chronic disease and its treatment is an important component of self-management, and studies have shown that poor disease knowledge is associated with higher health care services.^4^^,^^35^ In addition, it is noteworthy that a simple patient educational intervention, irrespective of the method, led to an improvement. However, increasing patient knowledge is only the first step in the process of activating patients to promote self-management. Thus, interventions may also focus on facilitating the patients with skills and confidence to enable self-management, which were described in 8 of the included studies.^15^^,^^20^^,^^22^^,^^23^^,^^25^^–^^29^

The delivery format of the interventions was different across studies, with the majority being in an outpatient setting, which was delivered remotely by nurses on an individual basis. In addition, the duration of the intervention varied. A systematic review and meta-analysis found that interventions with a psychological component in a group setting, led by health care professionals, and in <8 weeks duration showed the most beneficial effects for different health outcomes in connection to self-management.^36^ A multidisciplinary collaboration may be important to support the patients.^37^ Moreover, it should be taken into consideration how to ground the intervention and delivery within the clinical practice to prevent barriers from health care professionals to the implementation of self-management intervention.^38^

In the included studies, the intervention programs were all designed by the authors in connection with the individual studies, and only a few of the studies described the inspiration behind the program.^15^^,^^22^^,^^25^^–^^27^^,^^29^ Several self-management programs have been developed for chronic diseases, and their effects on health outcomes have been tested in randomized controlled trials. Thus, inspiration can be drawn from these programs when designing future self-management interventions for patients with cirrhosis. In addition, self-management is problem-based, and interventions must be based on patients’ needs and problems.^7^ Only few studies in this scoping review described that the interventions were developed to or tailored to the patients’ needs.^19^^,^^22^^,^^28^ Qualitative studies exploring self-management experiences in patients with liver disease have found that patients face uncertainties because of the provision of overly general self-management education.^39^ In addition, previous studies suggest that there is a divergence of focus between patients and health care professionals on what is important in connection with self-management. Health care professionals tend to focus on the complications and symptoms of cirrhosis disease or its prognosis, while patients’ places more focus on the limitations their disease imposes on their daily living, which impact their quality of life.^40^

To our knowledge, this is the first scoping review to review the literature on self-management interventions for patients with cirrhosis. The inclusion of intervention studies of various types provided a more wide-reaching review of current evidence, encompassing aspects such as the delivery format, description of the interventions, evaluation, and effect of the interventions. However, this also increased study heterogeneity, which limited comparison. This review included peer-reviewed articles published in English only found in 4 databases. In addition, there was no additional search for gray literature. Therefore, literature published in other databases, languages, or other websites may have been missed.

A formal assessment of the quality of the included studies is not required in scoping reviews as the aim of a scoping review is give an indication of the amount of literature available on a certain topic as well as an overview of its focus to identify key concepts and gaps.^41^ Seven of the included studies were randomized controlled trials.^15^^,^^19^^,^^20^^,^^22^^,^^23^^,^^26^^,^^27^ This design may eliminate some bias and thereby ensure internal validity. However, the other types of study design may pose a risk of bias that can affect the outcome. Therefore, the results of the included studies should be interpreted with caution. This may also be the reason why a systematic review and meta-analysis from 2020 assessing the clinical benefit of self-management programs for patients with cirrhosis found limited evidence of very low quality, indicating that the effect was very uncertain and the characteristics of self-management interventions in patients with cirrhosis were undefined.^3^

### Future directions for research and implications for clinical practice

Despite the limitations of this scoping review, it still provides an overview of the existing literature on self-management interventions to patients with cirrhosis to identify key concepts and gaps.

The studies included presented various study design, sample size, delivery format, self-management interventions designed by the authors, and evaluation. Although the interventions showed some improvement in patient-related and clinical outcomes, further longitudinal or randomized controlled trials are needed to assess the effect of self-management interventions. Moreover, the design of the self-management intervention should not only aim to facilitate the patients with knowledge, but also skills and confidences to increase self-efficacy and manage their disease. In that connection, it is crucial to measure the effect on the self-management intervention on both patient-related and clinical outcomes. Thus, multiple intervention strategies were needed to include the different tasks and skills required in connection to medical, behavioral, and emotional management. In this connection, it is an opportunity to draw inspiration from already tested self-management intervention programs for patients with chronic diseases and the included studies in this review. In addition, understanding the factors affecting self-management in patients with cirrhosis may help health care professionals provide better strategies for improvement. Therefore, health care professionals should assess the level of self-management in their patients and tailor the interventions to the patients’ needs to achieve positive outcomes (Figure 2).

## CONCLUSIONS

In conclusion, this scoping review highlights the benefits of self-management interventions for patients with cirrhosis. Despite the variation in study design, delivery format, self-management intervention, and evaluation, all included studies reported some positive patient-related and clinical outcomes, mainly within patient knowledge and quality of life. However, there is a need for more comprehensive and standardized self-management interventions tailored to patients´ needs, focusing on increasing confidence and self-efficacy and addressing the different tasks of self-management and skills required by the patients to manage their disease.
